# Organ donation: Key factors influencing the younger generation's decision-making in China

**DOI:** 10.3389/fpubh.2023.1052875

**Published:** 2023-02-06

**Authors:** Xiulan Chen, Wei Wei, Weili Ai

**Affiliations:** ^1^Department of International Finance, School of Finance, Shanghai Lixin University of Accounting and Finance, Shanghai, China; ^2^Shanghai Lixin University of Accounting and Finance, Shanghai, China; ^3^Department of Law, College of Philosophy, Law and Political Science, Shanghai Normal University, Shanghai, China

**Keywords:** organ donation, willingness, factor analysis, the younger generation, China

## Abstract

**Background:**

The organ transplantation sector in China is facing a severe shortage of donors, and the organ donation rate needs to be increased. Since 2015, voluntary donation by citizens has become the only source of organs for transplantation in China. In recent years, there has been a relatively positive change in young people's attitudes toward organ donation after death. The aim of the study was to understand young people's perceptions and attitudes toward organ donation and the factors that influence them and can positively impact the promotion of organ donation.

**Methods:**

By analyzing relevant literature and legal texts, we developed a questionnaire. Information was obtained through questionnaires and interviews, and 501 valid questionnaires were returned from the target group. A chi-square test was used to examine whether there were significant differences in the willingness to organ donation among young people with different characteristics. A factor analysis was used to investigate the main factors influencing the different attitudes of young people toward organ donation, and a one-way ANOVA was used to examine whether young people with different characteristics were affected differently by different factors.

**Results:**

In our survey of young people aged 18–30 years, 99.2% of respondents knew about organ donation, 47.1% were willing to donate organs, and 15.2% understood that there were corresponding laws and regulations for organ donation. The study's findings showed that urban residents are more willing to be organ donators than rural residents; people with higher education levels have better awareness and are more willing to donate an organ; and people with religious beliefs are more likely to donate organs. The main factors that support the willingness of young people to donate are the social environment that provides support, their optimism in dealing with death, and their desire to realize their final value after death. The main factors for those unwilling to donate were low awareness or misconceptions about organ donation among individuals and their families and their attitudes toward death. As the people who took the questionnaire are probably interested in organ donation, the sample results will show a higher percentage of people who know about organ donation. We hope to discuss further with a larger and broader sample coverage to improve the estimates' validity and reflect the overall picture more accurately in a future study.

**Conclusion:**

Young people knew about organ donation but had a low depth of awareness. Household registration type, education level, and religious affiliation significantly correlate with people's willingness to donate. The supportive environment for organ donation in society and the correct understanding of the organ donation process and laws and regulations can influence people's willingness to donate.

## 1. Introduction

Organ transplantation is one of the most outstanding achievements of the 20th century. For patients suffering from end-stage organ diseases, organ transplantation gives them a chance to be “reborn.” As the technology of organ transplantation gradually improved, the topic of organ donation has become a hot issue over the last decade in China. According to the National Health Commission of China, both the number of organ donations and the number of transplants in China have increased significantly from 2015 to 2018. In China, 2,766, 4,080, 5,146, and 6,302 organ donations were completed each year from 2015 to 2018. The number of organ transplants in China exceeded 20,000 in 2018, ranking second in the world regarding the number of operations performed. Furthermore, the survival rate of recipients after organ transplantation in China has reached an internationally advanced level. In 2018, the 1 and 3-year cumulative survival rates of recipients after living liver transplantation in China were 92.5 and 89.8%, respectively, which were close to the 1 and 3-year cumulative survival rates of recipients after living liver transplantation in the United States (92.3 and 88.4%). In 2018, the 1-year survival rate after heart transplantation in China was 90.8%, and the 1 and 3-year survival rates of recipients after kidney transplantation were 96.7 and 95.6%, both at the advanced international level. According to the latest data released by the China Organ Donation Management Center, as of 30 May 2021, 34,245 organ donors donated, and the number of registered organ volunteers in China was 3,296,260. As of 2020, the National Health Commission of China has recorded 170 medical institutions with qualifications for human organ transplantation and 33 training bases for human organ transplantation.

Although the number of those requiring organ transplants has grown rapidly, the number of those registered to donate organs remains very insufficient in comparison. According to IRODaT data (International Registry in Organ Donation and Transplantation, available at www.irodat.org), in 2021, the worldwide actual deceased organ donors' rate (number of donors per million people, “pmp” for short), the United States ranked first, 41.88; the United Kingdom, 20.12; and China, 3.63, and the worldwide living organ donors' rate (pmp), Turkey ranked first, 51.92; the United States, 19.75; the United Kingdom, 11.88; and China, 2.25. The gap between China and other countries is huge. In recent years, with the promulgation of a series of documents and norms such as the WHO Guidelines for Human Cell, Tissue and Organ Transplantation, the Declaration of Helsinki, the Regulations on Human Organ Transplantation in China, the Basic Principles of Human Organ Allocation and Sharing, and the Core Policy on Liver and Kidney Allocation and Sharing in China (Version 2010), organ donation has become much more standardized in China. People's awareness of organ donation has also increased. There is also a growing awareness and understanding of organ donation.

The Regulations on Human Organ Transplantation in China, which came into effect on 1 May 2007, stipulate that human organ donation shall be based on the principles of voluntariness and gratuitousness. Citizens have the right to donate or not donate their organs; no organization or individual may force, deceive, or induce others to donate their human organs. Voluntary organ donation in China began in 2010. Since 2015, voluntary donation by citizens has become the only source of organs for transplantation in China.

From December 2020 to April 2021, we surveyed the public's perceptions and attitudes toward organ donation in Chinese society, and this survey included people of all ages. This previous survey showed that the younger Chinese generation (18–30 years old) has a positive perception of organ donation, and the influencing factors are significantly different from the older generation. The aim of the study was to understand young people's perceptions and attitudes toward organ donation and the factors that influence them and can positively impact the promotion of organ donation.

## 2. Literature review

Regarding organ donation, most researchers currently focus on the following points: the public's knowledge about organ donation and their willingness to donate their organs, the key factors influencing families' decision-making regarding organ donation, and the rights of human organs and the ethical controversy of organ donation.

Since 2010, several studies have been conducted to investigate public attitudes toward organ donation in China (1, 2). Lei et al. (3), Liang et al. (4), Zhang et al. (5), and Long and Liu (6) specially selected a group of college students to study the young generation's attitude toward organ donation (3–6). Hu (7) conducted separate surveys on medical workers, medical students, and the general public to compare the differences in the perceptions, attitudes, and willingness to donate organs among these three groups in a cross-sectional manner (7). Lei et al. (3) administered questionnaire surveys to evaluate the different perspectives of medical students and non-medical students toward organ donation (3). In the United States, the study by Hafzalah assessed American Muslims' willingness to donate organs (8). They found that a lack of awareness of the support of Islam for organ donation and the fear of disfigurement have negatively affected organ donation willingness in Muslim communities. Kobus et al. studied the attitudes and opinions of Judaism's followers regarding organ donation (9). The results showed that most Jewish believers were willing to accept organ transplantation. More than 90% of those interviewed had a positive attitude toward organ transplantation. Kapikiran et al. investigated the knowledge and attitudes about organ donation from the perspective of liver transplant patients (10). The majority of respondents were willing to receive an organ donation as well as to donate an organ. Eventually, all the above studies will agree that awareness should be raised in society about organ donations.

Regarding the willingness of donor family members to donate organs and influencing factors, several studies focused on the key factors influencing families' decision-making regarding organ donation after the death of their family member (11, 12). They analyzed why parents of potential child donors declined to donate organs (13). These surveys showed that fear of surgical pain, disfigurement, and local customs are the main reasons parents of potential child donors refuse organ donation. A special study in Northwestern China concluded that popularizing organ donation knowledge and establishing reasonable compensation and incentive mechanisms may effectively increase the organ donation rate in the economically underdeveloped regions of China (14).

Zeng et al. (15) believed that critically ill patients were often potential organ donors; they surveyed the families of critically ill patients regarding their willingness to donate organs (15). The results showed that measurements such as reinforcement of propaganda education, the incentive of altruistic behavior, and improvement of compensation and legislation systems might enhance public willingness to donate an organ. Zhang et al. (16) and Sun et al. (17) investigated local community residents' knowledge and attitudes toward organ donation. They found that among the many factors that affect people's willingness to donate organs, traditional Chinese cultural values are an essential factor (16, 17). Lin et al. (18) also found that family opposition is the biggest roadblock hindering organ donation (18). Qian et al. (19) also found that the main reason potential donors fail to register to donate an organ was family disapproval of such a donation (19). Flemming et al. (20) studied African-American perceptions of organ donation. They found that respondents were more likely to donate organs if they understood the pros and cons of organ donations (20).

Jurisprudents are more concerned about the rights of human organs, and social scholars are concerned about the ethical issues of organ donation. Wang (21) discussed the dual attributes of the rights of human organs and believed that the rights of human organs should not be artificially divided into two rights with entirely different attributes just because of “their separation from the human body” (21). Gong (22) proposed that in human organ transplantation, a legal system and an operational mechanism should be created which meet reality and maximize the life and health needs of human organ recipients under the premise of respecting the willingness of organ providers and under the constraints of the theories and jurisprudential principles of balanced protection of human rights, differentiation of value and interest levels, respect for life ethics, and balance of fairness and effectiveness concerning rights and interests (22). Li (23) discussed the liability for repentance and damage compensation for organ donation and argued that to avoid the arbitrary exercise of revocation and repentance by organ donors, which made the recipient an innocent victim in the act of repentance, the liability for compensation and damage compensation when the organ donor's repentance causes damage to the recipient needed to be determined (23). Chandler et al. investigated public reactions to giving prioritization in organ allocation to previously registered donors (24). Supporters justify priority systems because they are fair and will encourage donor registration. There are concerns about the social division that the priority system may cause. Li et al. explored the ethical review and supervision system of organ donation before and after citizen death in medical institutions (25). Yu et al. discussed the concept of incentives in organ donation. They provided an overview of regulations on organ donation by international organizations, focusing on the ethical issues involved in organ donation research and management (26). Luo et al. found that organ donor families were in desperate need of material and emotional support (27).

Although a series of policies have been issued worldwide to promote organ donation, the actual number of organ donations needs to meet the huge demand for organs in China, and the construction of the field of organ donation is in the initial stage of development. Problems such as the low rate of public organ donation, irregular organ donation procedures, incomplete laws and regulations, and illegal organ trading exist in China. We need more surveys to clarify the underlying factors influencing the willingness to donate organs and enhance public awareness and support of organ donation.

## 3. Material and methods

Random samplings of 501 respondents consisting of young Chinese people aged 18–30 years were selected for this study. In China, “18–30 years old” is called “post-90's” and “post-00s.” It refers to young people born after 1990. They were maybe in the late stages of high school, university, or doctoral stage or had worked for a few years. From December 2020 to April 2021, we conducted the survey mainly through online and offline questionnaires.

Before the formal distribution of the questionnaire, we randomly selected 20 people to ask questions such as “whether the questionnaire questions are easy to understand” and “whether the answers to the questionnaire contain all possible answers,” revised them according to their opinions, and then distributed the revised formal questionnaire. A total of 600 questionnaires were distributed during the standard survey, and 501 questionnaires were completed and submitted, which is an 83.5% response rate. The same 20 people also filled out the standard questionnaire in the follow-up and became part of the 501 samples.

In terms of sampling, due to geographical constraints, our offline survey was mainly focused on Eastern China, with the majority of the sample coming from online. A small number of questionnaires were also distributed offline in case the respondents did not understand the questions, and we were able to deal with them in time. Fortunately, with the help of the previous 20 interviewers, no such situation was found offline. There are several online channels: the first is a questionnaire website, Questionnaire Star (a well-known survey website in China, www.wjx.cn), which has a corresponding incentive system to ensure the distribution and return of questionnaires, and it also sends out questionnaires by the principle of randomness. The second way is through our social networking software, such as WeChat, QQ, and Weibo (similar to Twitter), to distribute the questionnaires like advertisements. The questionnaire is usually filled out by interested people who meet the age requirement. Online distribution means that the questionnaire is opened, but only when it is completed and submitted does it mean that we have received feedback. By the end of 2021, WeChat, QQ, and Weibo had 1.268 billion, 552 million, and 573 million monthly active users, respectively. Therefore, questionnaires distributed *via* WeChat, QQ, and Weibo will reach most of China's young generation. In the case of Weibo, which is similar to Twitter, for example, a tweet posted by a celebrity is usually viewed by millions. Of course, the people who responded to the questionnaire include many people we know, but many more are people we do not know. From our survey results, the sample profile's diversity is also very diverse. It would be better to reach a more comprehensive sample and a wider range of online and offline people. We had a goal to collect at least 400 questionnaires within a month, so when we collected 501 questionnaires within a month (600 questionnaires were distributed), we thought the samples were basically enough.

We stratified them according to the nature of their household registration, gender, education level, and religious belief. In general, there are two forms of expression of willingness to donate organs in China:

(1) Citizens have expressed their willingness to donate organs for free and have registered with a qualified institute;
(2) Citizens have not expressed their unwillingness to donate before their deaths, but their relatives and family members jointly agreed to donate for free after the citizen's death.

Based on these two points, the core questions of this survey are “When your life cannot be saved, are you willing to donate organs?”, “Have you ever registered for organ donation?”, and “When your loved ones' lives cannot be saved, are you willing to donate organs as their family member?”.

Through the interviews with a small group of people in our pre-survey, it was found that the interviewees who are willing to donate generally have a good understanding of organ donation. Less influenced by traditional Chinese concepts like “keeping the whole body” and “going to the soil for safety,” they consider organ donation a great contribution to society by saving the lives of others and recognize its value in the development of medical care and research.

Therefore, we designed the questionnaire considering the influencing factors as much as possible for a survey to understand the source of the motivation of “willing to be a donor.” Among the small group interviewees, we found that people unwilling to donate generally have low awareness and know little about organ donation. They have no idea about the process of organ donation. Most of them have severe concerns about organ abuse. The “unwillingness to donate” group and the issue should be investigated from different angles.

The overall content of the questionnaire was set up around six aspects of organ donation: the basic information of the interviewees, awareness of organ donation, attitudes toward organ donation, motivation, underlying reasons for reluctance to donate, and problems that need to be solved urgently in organ donation and possible countermeasures.

The data analysis included the chi-square test, factor analysis, and a one-way analysis of variance (ANOVA). The chi-square test was used to study whether the public with different characteristics has significant differences in organ donation choices, and the factor analysis was used to study groups with varying attitudes toward donation. The main influencing factor, a single-factor analysis of variance, was used to investigate whether the public with different characteristics is affected by other factors to varying degrees. Collected data were analyzed using SPSS, version 22.0 (IBM SPSS).

## 4. Analysis of results

### 4.1. Background information

The detailed sociodemographic characteristics of the study participants are mentioned in Table 1. The “household registration” was divided into “rural resident” and “urban resident.” Because our presurvey showed a significant difference in organ donation willingness between urban and rural residents regarding gender, the proportions of men and women selected for the survey are not much different. In terms of education, many respondents had undergraduate and junior college degrees.

**Table 1:** Sociodemographic characteristics of respondents of the survey.

	**Type**	**Frequency**	**Percentage**
Household registration	Urban	386	77.0
	Rural	115	23.0
Gender	Male	172	34.3
	Female	329	65.7
Education level	Junior high school and below	13	2.6
	High school and technical secondary school degree	17	3.4
	Bachelor's and college degree	460	91.8
	Postgraduate and above	11	2.2
Religious belief	Have religious beliefs	15	3.0
	No religious belief	486	97.0

In 2020, the ratio of male population to female population in China was 51.2:48.8 (according to the National Bureau of Statistics of China). We have tried our best to consider the gender issue comprehensively in this survey. However, unfortunately, we received more responses from women. We suspect that it is likely that more women are registered on Questionnaire Star, and it is also likely that our team is mostly composed of women. On WeChat and QQ, most of our contacts are women, more questionnaires were delivered to women, and as a result, the sample is composed of more women. This is one of our limitations, and we hope to improve it in future studies.

We separately investigated sample groups of religious and non-religious persons to investigate whether the two groups have a different willingness to donate organs. In Table 1, the basic information of the various types of respondents is distinguished in detail. The purpose of the distinction is to examine the public's awareness of and willingness to donate an organ and the factors affecting their willingness to contribute under different circumstances.

### 4.2. Analysis of the public's knowledge and attitude toward organ donation

#### 4.2.1. Knowledge and attitude toward organ donation

Table 2 shows that the respondents have a relatively high awareness of organ donation. Most of them learned about organ donation and understood its meaning through media such as radio, television, and the Internet. But they have low awareness of the specific laws and regulations in the organ donation area. However, more than 40% of respondents (young people aged 18–30 years) have a strong willingness to be organ donors. This result shows that the current public's attitudes have changed significantly compared to our previous survey's results. Although the importance of organ donation and transplantation is widely recognized, many respondents who are willing to donate have not registered to donate organs. Many of those who are willing to donate but have not registered feel they have not discussed it with their families, do not know if their families will oppose them, are not sure if their attitudes will change with outside intervention, or are unfamiliar with the organ donation process. After all, they recognize the necessity of an organ donation incentives policy, such as a priority system for organ transplantation and medical protection. At the same time, they are unfamiliar with relevant procedures for organ donation.

**Table 2:** Respondents' awareness of organ donation.

	**Category**	**Frequency**	**Percentage**
Do you know about organ donation?	Yes	497	99.2
	No	4	0.8
Do you know the laws and regulations on human organ transplantation in China?	Yes	76	15.2
	No	425	84.8
Do you know that China remarkably lacks organ donation?	Yes	410	81.8
	No	91	18.2
Do you know the current sources of transplanted organs?	Yes	184	36.7
	No	317	63.3
Are you willing to donate organs after death?	Yes	236	47.1
	No	265	52.9
Have you filled in the organ donation registration form?	Yes	9	1.8
	No	492	98.2
Do you support organ donation registration when applying for a driver's license?	Yes	74	14.8
	No	311	62.1
	Neutral	116	23.2
Do you support the practice of a priority system for organ transplantation and medical protection, etc.?	Yes	383	76.4
	No	46	9.2
	Neutral	72	14.4

#### 4.2.2. Comparative analysis of different public attitudes toward organ donation

To compare the public's attitudes toward organ donation, we used background information as the independent characteristic variables such as household registration, gender, age, education level, and religious beliefs to conduct a chi-square test (see Table 3).

**Table 3:** Background information and organ donation awareness.

**Background information**	**Type**	**Are you willing to donate organs after death?**		**Pearson's chi-squared test**	**Progressive sig. (Both sides)**
		**Yes (236)**	**No (265)**		
Household registration	Urban	191 (49.5%)	195 (50.5%)	3.810	0.051^*^
	Rural	45 (39.1%)	70 (60.9%)		
Gender	Male	87 (50.6%)	85 (49.4%)	1.270	0.260
	Female	149 (45.3%)	180 (54.7%)		
Educational level	Junior high school and below	0 (0.0%)	13 (100.0%)	24.928	0.000^***^
	High school and technical secondary school degree	6 (35.3%)	11 (64.7%)		
	Bachelor's and college degree	219 (47.6%)	241 (52.4%)		
	Postgraduate and above	11 (100.0%)	0 (0.0%)		
Religious belief	Have religious beliefs	13 (86.7%)	2 (13.3%)	9.713	0.002^***^
	No religious belief	223 (45.9%)	263 (54.1%)		

^***^, ^**^, and ^*^ indicate significance at the 1, 5, and 10% significance levels, respectively.

The assumptions are as follows:

Null hypothesis 1: There is no significant difference in the willingness to donate organs among the public with different household registration;
Null hypothesis 2: There is no significant difference in the willingness to donate organs of the public in different genders;
Null hypothesis 3: There is no significant difference in the willingness to donate organs among the public with different education levels;
Null hypothesis 4: There is no significant difference in the willingness to donate organs of the public with or without religious beliefs.

Since three-fourths of the null hypotheses are rejected (see Table 3), the following conclusions can be summarized:

(1) There is a significant difference in the choice of organ donation among the public with different household registrations. The proportion of rural residents unwilling to donate is higher than that of urban residents.
(2) No significant difference in the choice of organ donation is found among the general public of different genders. From the 501 samples, 87 of 172 men, or 50.6%, were willing to donate organs, and 149 of 329 women, or 45.3%, were willing to donate organs. Although men were more likely to donate organs than women, according to the sample data, the chi-square test reveals that this difference was not significant in the inferred overall population.
(3) There is a significant difference in the choice of organ donation among the public with different levels of education. According to our interview, the respondents with higher education levels have better awareness and a stronger willingness to donate an organ.
(4) There is also a significant difference in the choice of organ donation between the religious and non-religious public. According to our survey results in China, the religions of the respondents are mainly Buddhism and Taoism. These religions advocate a more open-minded attitude toward life and death. Therefore, believers are more supportive of organ donation than those without religious beliefs.

### 4.3. Factor analysis

The above results showed significant differences in attitudes toward organ donation among respondents from different backgrounds. The key factors that influence their decision to donate are complicated. To further analyse the influencing factors, the exploratory factor analysis method was used to classify each influencing factor and examine the degree of its influence on the attitudes of the respondents.

#### 4.3.1. Descriptive statistics of influencing factors

Table 4 shows the scores of 236 respondents who are willing to donate organs. The average value is above 3 (5-level scale). It means that these factors can affect the respondents' decision-making for donation. Some factors, such as “facing death calmly,” “the society's advocacy for dedication,” and “organ transplantation is a way to extend one's own life,” have greater influence. This item has the highest average score and the smallest standard deviation, indicating that the sample population is affected by the item with the strongest average value and tends to be consistent.

**Table 4:** Descriptive statistics (willingness to donate organ).

	**Mean**	**Std. deviation**	**Analysis *N***
X1: Facing death calmly	4.4576	0.85197	236
X2: Rescue others and realize the ultimate value of oneself	3.9831	1.04766	236
X3: Organ transplantation is a way to extend one's own life	4.5466	0.80023	236
X4: Promote the development of the medical industry	3.7966	1.08811	236
X5: Support from family members and friends for organ donation	3.8856	1.19201	236
X6: Education on organ donation	3.9534	1.04064	236
X7: The society's advocacy for dedication	4.0551	1.02373	236
X8: Awareness of the organ donation process	3.8983	1.09459	236
X9: Awareness of laws and regulations on organ donation	3.8602	1.12682	236
X10: Knowledge of the organs transplantation process	3.9237	1.10824	236
X11: Donors and relatives can get priority right to be donated	3.8898	1.13950	236
X12: Families can get better humanitarian assistance	3.6483	1.20955	236

Table 5 shows that the 265 respondents who are unwilling to donate have relatively high scores on the degree of influence for each factor. The average value is also above 3 (5-level scale). The respondents thought that the following items could significantly affect their decision: “avoidance behaviors toward life and death issues,” “family disapproval,” “family members do not know the laws and regulations on organ donation,” and “fear of organ transplantation abuse.” The deviations of these items are relatively small, which means the respondent's attitude tends to be consistent.

**Table 5:** Descriptive statistics (unwillingness to donate organ).

	**Mean**	**Std. deviation**	**Analysis *N***
Y1: Avoidance behaviors toward life and death issues	3.8792	1.03378	265
Y2: Traditional concept of “keeping the whole corpse”	3.9509	1.02315	265
Y3: Family disapproval	4.0340	1.01633	265
Y4: Family members don't know the laws and regulations on organ donation	4.1774	0.96268	265
Y5: Insufficient media promotion	3.5434	0.91235	265
Y6: Unfair use related to a priority system	3.4830	1.08043	265
Y7: Lack of death certificate	3.0792	0.99874	265
Y8: Lack of laws and regulations on organ donation	3.5698	1.24766	265
Y9: Worry about the credibility of the donation system	3.7547	1.09934	265
Y10: Worry about organ transplantation abuse	4.1019	1.03764	265

#### 4.3.2. Reliability and validity test

Cronbach's α coefficient was used for the reliability test. In general, Cronbach's α coefficient above 0.65 is the minimum acceptable reliability value. Table 6 shows that the influencing factors “willingness to be an organ donor” and “unwillingness to be an organ donor” are both >0.8, which belongs to a very reliable interval range and can pass the reliability test.

**Table 6:** Reliability statistics.

**Influence factor**	**N. of items**	**Cronbach's alpha**
Factor influencing willingness to donate organs	12	0.998
Factor influencing unwillingness to donate organs	10	0.997

The KMO and Bartlett sphericity tests were used for the validity test. The KMO test is based on the comparison of simple correlations and partial correlations between different variables. In general, if KMO > 0.9, the data obtained are very suitable for factor analysis; if KMO > 0.7, the data obtained are more suitable; and if KMO <0.5, the data obtained are not suitable for factor analysis. The Bartlett sphere test tests whether the correlation matrix is an identity matrix or whether each variable is independent. According to Tables 7, 8, the KMO value is >0.7, and the probability value of the significance test is smaller than 0.05. It means that the results can pass the validity test and are suitable for factor analysis.

**Table 7:** KMO and Bartlett's test (willingness to donate organ).

	**Kaiser-Meyer-Olkin measure of sampling adequacy**		**0.850**
Bartlett's Test of sphericity	~Chi-Square	2,285.420
	Df	66
	Sig.	0.000

**Table 8:** KMO and Bartlett's test (unwilling to donate organ).

**Kaiser-Meyer-Olkin measure of sampling adequacy**		**0.728**
Bartlett's test of sphericity	~Chi-Square	1,756.346
	Df	45
	Sig.	0.000

#### 4.3.3. Exploratory factor analysis

Many factors influence the public's attitude toward organ donation, and the relationships among the affective factors are complicated. The KMO and Bartlett's sphericity test show that these factors have a strong correlation and can be summarized into several representative items. To study these factors as independent variables, we constructed a multifactor model to extract the main factors that affect the “willingness to donate organs” group and the “unwillingness to donate organs” group, respectively, and to find out some common characteristics among these influencing factors.

We assume that there are *n* main factors influencing the willingness to donate *X*_1_,……*X*_*n*_. Through factor analysis, these *n* original variables are expressed as the linear weighted sum of *k* common factors (*f*_1_,*f*_2_ ,……*f*_*k*_) (*k*≤*n*) and a special factor ε_*i*_. The principal component method was used to extract the common factors, and the matrix expression is as follows:


(1) $$\begin{array}{c} \begin{array}{c} \left[\begin{array}{c} X_{1}\\ X_{2}\\ ...\\ X_{n} \end{array}\right]=\left[\begin{array}{cccc} \alpha_{11} & \alpha_{12} & ... & \alpha_{1k}\\ \alpha_{21} & \alpha_{22} & ... & \alpha_{2k}\\ ... & ... & ... & ...\\ \alpha_{n1} & \alpha_{n2} & & \alpha_{nk} \end{array}\right]\;\;\left[\begin{array}{c} f_{1}\\ f_{2}\\ ...\\ f_{k} \end{array}\right]+\left[\begin{array}{c} \varepsilon_{1}\\ \varepsilon_{2}\\ ...\\ \varepsilon_{n} \end{array}\right] \end{array} \end{array}$$

We used the maximum variance method to obtain the explained total variance (as shown in Tables 9, 10). We then extracted the main factor according to the factor extraction criterion with a feature value >1 (as shown in Tables 11, 12).

**Table 9:** Total variance explained (willingness to be an organ donor).

**Component**	**Initial eigenvalues**			**Extraction sums of squared loadings**			**Rotation sums of squared loadings**		
	**Total**	**% of** **variance**	**Cumulative** **%**	**Total**	**% of** **variance**	**Cumulative** **%**	**Total**	**% of** **variance**	**Cumulative** **%**
1	6.340	52.837	52.837	6.340	52.837	52.837	4.392	36.602	36.602
2	1.506	12.551	65.388	1.506	12.551	65.388	3.267	27.224	63.825
3	1.100	9.163	74.550	1.100	9.163	74.550	1.287	10.725	74.550

**Table 10:** Total variance explained (unwillingness to be an organ donor).

**Component**	**Initial eigenvalues**			**Extraction sums of squared loadings**			**Rotation sums of squared loadings**		
	**Total**	**% of variance**	**Cumulative %**	**Total**	**% of variance**	**Cumulative %**	**Total**	**% of variance**	**Cumulative %**
1	4.744	47.443	47.443	4.744	47.443	47.443	3.293	32.933	32.933
2	1.561	15.613	63.055	1.561	15.613	63.055	2.233	22.334	55.267
3	1.009	10.088	73.144	1.009	10.088	73.144	1.788	17.876	73.144

**Table 11:** Rotated component matrix (willingness to be an organ donor).

	**Factor**		
	**f1**	**f2**	**f3**
X1: Facing death calmly	0.119	**0.827**	0.102
X2: Rescue others and realize the ultimate value of oneself	−0.074	0.290	**0.797**
X3: Organ transplantation is a way to extend one's own life	0.070	**0.842**	0.171
X4: Promote the development of the medical industry	0.510	−0.012	**0.646**
X5: Support from family members and friends for organ donation	**0.669**	0.602	0.108
X6: Education on organ donation	**0.706**	0.477	−0.083
X7: The society's advocacy for dedication	**0.662**	0.317	−0.144
X8: Awareness of the organ donation process	**0.693**	0.598	0.147
X9: Awareness of laws and regulations on organ donation	**0.663**	0.623	0.031
X10: Knowledge of the organs transplantation process	**0.698**	0.570	0.113
X11: Donors and relatives can get priority right to be donated	**0.856**	0.081	0.154
X12: Families can get better humanitarian assistance	**0.765**	−0.152	0.310

Bold values indicate the factor with the highest load of each X.

**Table 12:** Rotated component matrix (unwillingness to be an organ donor).

	**Factor**		
	**f1**	**f2**	**f3**
Y1: Avoidance behaviors toward life and death issues	0.184	**0.939**	0.157
Y2: Fear of deformity in the corpse	0.122	**0.947**	0.137
Y3: Family disapproval	**0.564**	0.371	0.112
Y4: Family members don't know the laws and regulations on organ donation	**0.802**	0.252	0.134
Y5: Insufficient media promotion	0.438	0.119	**0.737**
Y6: Unfair use related to the priority system	0.083	0.202	**0.896**
Y7: Lack of death certificate	**0.727**	−0.039	0.125
Y8: Lack of laws and regulations on organ donation	**0.759**	0.409	0.059
Y9: Worry about the credibility of the donation system	**0.747**	−0.060	0.472
Y10: Worry about organ transplantation abuse	**0.649**	0.167	0.355

Bold values indicate the factor with the highest load of each Y.

In general, if the variance explained by each factor extracted is not very different and the cumulative variance interpretation rate is more than 60%, then the factor analysis extraction effect is better. Tables 9, 10 show that when factors are extracted from the influencing factors of “wish to donate” and “unwilling to donate,” the cumulative variance contribution rate meets the requirements. Using the extraction factor score to analyse the influencing factors of organ donation willingness, the comprehensive evaluation model obtained is as follows:

For those who are willing to donate:


(2) $$\begin{array}{c} f=\frac{36.602}{74.550}f_{1}+\frac{27.224}{74.550}f_{2}+\frac{10.725}{74.550}f_{3} \end{array}$$

For those who are unwilling to donate:


(3) $$\begin{array}{c} f=\frac{32.933}{73.144}f_{1}+\frac{22.334}{73.144}f_{2}+\frac{17.876}{73.144}f_{3} \end{array}$$

Table 11 shows the load on different variables of the three factors in the group “willingness to be an organ donor.” Because it focuses on different specific angles, it is named based on its meaning: Factor 1 has a large load on the variables *X*_5_−*X*_12_, and mainly focuses on the external social atmosphere faced by the donor. According to its meaning, it is named “the social atmosphere factor.” Factor 2 has a larger load on variables *X*_1_and *X*_3_ and focuses on the donor's own ideas, so it is named “the personal perception factor.” Factor 3 has a larger load of variables, *X*_2_ and *X*_4_ focuses on the influence of the incentive mechanism on the willingness to donate, so it is named “altruism factor.” The results show that the sample groups' willingness to donate is most affected by the social atmosphere, personal perceptions, and altruism.

Table 12 shows the load on different variables of the three factors for those who are unwilling to be organ donors. Factor 1 has a large load on the variables *Y*_3_−*Y*_4_, *Y*_7_−*Y*_10_ concerning the local organ donation system, including the death certificate process, the organ transplantation process, and the fairness of organ distribution. Therefore, it is named “the cognitive bias factor” based on its meaning. Factor 2 has a large load on the variables *Y*_1_−*Y*_2_, related to their family members and own concerns toward organ donation. Therefore, we named it the “traditional stereotype factor.” Factor 3 has a relatively large load on the variables *Y*_5_−*Y*_6_. The respondents worried that if donated organs are used for transplantation, they will be given priority to people of higher social class, while those of lower social class may not be able to afford medical expenses and have no opportunity to receive organ transplants. Therefore, we named it the “fairness concern factor.”

### 4.4. Analysis of impact factors on public attitudes toward organ donation

The above exploratory factor analysis method regrouped many factors affecting the public's attitude toward organ donation into three principal factors. Then, the one-way analysis of variance method was used to analyse the different levels of factors' impact. The results are shown in Tables 13, 14.

**Table 13:** ANOVA (willingness to be an organ donor).

**Impact factors and background information**	**F**	**Sig**.
Household registration and social atmosphere	0.003	0.958
Household registration and personal perception	10.137	0.002^***^
Household registration and policy incentives	1.407	0.237
Gender and social atmosphere	0.100	0.752
Gender and personal perception	18.436	0.000^***^
Gender and policy incentives	0.336	0.563
Educational level and social atmosphere	5.726	0.004^***^
Educational level and personal perception	0.424	0.648
Educational level and policy incentives	5.806	0.003^***^
Religious beliefs and social atmosphere	11.300	0.001^***^
Religious beliefs and personal perception	0.421	0.517
Religious beliefs and policy incentives	6.802	0.010^***^

^***^, ^**^, and ^*^ indicate significance at the 1, 5, and 10% significance levels, respectively.

**Table 14:** ANOVA (unwilling to be an organ donor).

**Impact factors and background information**	**F**	**Sig**.
Household registration and fairness concern factor	15.119	0.000^***^
Household registration and family resistance factor	1.259	0.263
Household registration and cognitive biases factor	2.350	0.126
Gender and fairness concern factor	3.027	0.083^*^
Gender and family resistance factor	0.365	0.546
Gender and cognitive biases factor	15.449	0.000^***^
Educational level and fairness concern factor	0.976	0.378
Educational level and family resistance factor	5.613	0.004^***^
Educational level and cognitive biases factor	0.975	0.379
Religious belief and fairness concern factor	2.106	0.148
Religious belief and family resistance factor	2.511	0.114
Religious belief and cognitive biases factor	0.181	0.671

^***^, ^**^, and ^*^ indicate significance at the 1, 5, and 10% significance levels, respectively.

For those who were willing to donate, the influence of the “social atmosphere factor” was significant at the 1% level of significance for respondents with different levels of education and religious beliefs, indicating that there was a significant difference in their influence on this factor; the influence of the “optimism factor” was significantly different for respondents with different natures of household registration and gender, and the original hypothesis of “no significant difference in influence” was rejected at the 1% level of significance; the influence of the “value realization factor” was significantly different for respondents with varying levels of education and religious beliefs and for those with no religious beliefs.

For those who are unwilling to be organ donors, the respondents with different ages and household registration are mainly affected by the “cognitive biases factor.” Due to different sociodemographic characteristics such as household registration, gender, education level, and religious belief, the impacts of the “family resistance factor” and “fairness concern factor” are significantly different on the willingness to donate.

## 5. Limitations

We wanted to find out about the willingness of China's younger generation to donate organs by surveying our sample, and we did so according to the principle of random sampling. Due to the limitations of our survey, the sample deviated from the total. First, our offline survey was mainly conducted in Eastern China, so the sample results will show more characteristics of the young generation in that region. Second, as the people who took the questionnaire, including those on Questionnaire Star and those on social networking sites, are probably interested in organ donation, the sample results will show a higher percentage of people who know about organ donation. Third, as more women follow us on our social networking software, the response rate is also higher for women. The literature clearly shows that women are more interested in this type of social issue than men, which is reflected to some extent in the higher proportion of women in the sample.

Although we have reduced the impact of these limitations on the study by stratifying the sample with factors such as gender and age, in future studies, we still hope to improve this issue of randomness with a larger and broader sample coverage to improve the validity of the estimates and to reflect the overall picture more accurately.

## 6. Conclusion

The problem of insufficient organ donation in China and worldwide has seriously affected the development of organ transplantation. Promoting organ donation and increasing organ availability are necessary to save the lives of patients who need a transplant and shorten the waiting time on the waiting list.

We differ from other articles in that many of them do not investigate the reasons for willingness and unwillingness to donate organs separately; some only consider family factors, and some only consider social factors, whereas we have set a number of topics for the reasons, trying to find deeper reasons that come from the researcher's understanding of Chinese reality and from his research on the issue before starting this study. Regarding awareness of organ donation, in our survey of young people aged 18–30 years, we found that 99.2% of respondents knew about organ donation, 47.1% were willing to donate organs, and 15.2% understood that there were corresponding laws and regulations for organ donation. Overall, young people knew about organ donation but had a low depth of awareness. Regarding differences in attitudes toward organ donation: a chi-square test was performed on the sample data, and we find that urban residents are more willing to be organ donors than rural residents; no significant difference in the choice of organ donation is found among the general public of different genders; people with higher education levels have better awareness and are more willing to donate an organ; people with religious beliefs are more likely to donate organs. By using exploratory factor analysis, we find that the factors that support young people's willingness and unwillingness to donate also differ. The main factors that support the willingness of young people to donate are the social environment that provides support, their optimism in dealing with death, and their desire to realize their final value after death. The main factors for those unwilling to donate were low awareness or misconceptions about organ donation among individuals and their families and their attitudes toward death.

To promote organ donation among the young generation, China needs to reconsider the role of families in the decision-making process. It is essential to increase organ donation awareness among the younger generation and encourage them to discuss their willingness to donate with their families. In addition to enhanced publicity, there is also a need to improve policies on humane care and incentives for organ donation.

## Data availability statement

The raw data supporting the conclusions of this article will be made available by the authors, without undue reservation.

## Ethics statement

Ethical review and approval was not required for the study on human participants in accordance with the local legislation and institutional requirements. The information we used for the study was obtained through questionnaires and interviews. When respondents completed the questionnaire or were interviewed, we clearly communicated the purpose of the survey and their completion of the questionnaire and/or acceptance of the interview implied their consent to participate in this survey.

## Author contributions

XC conceived the main ideas of the paper, especially the econometric part, led the whole paper, and contributed much to each part. WW joins every aspect of the paper, especially the wording and tables. WA has expertise in law, and his professional competence and professional work ensured the integrity of our thesis, contributed a lot to every part of the paper.
