# Supplementary material for: Organ donation: Key factors influencing the younger generation's decision-making in China
Source: Front Public Health. 2023 Feb 6;11:1052875. doi: 10.3389/fpubh.2023.1052875 (PMC9940821; doi:10.3389/fpubh.2023.1052875)
Supplement: Supplementary file 1 [file Data_Sheet_1.pdf]

社会公众对器官捐献的态度、动机及影响因素

Public attitude, motivation and influencing factors towards organ donation

保密：根据《中华人民共和国统计法》第三章第二十五条，“统计调查中获得的能够识别或者推断单个统计调查对象身份的资料，任何单位和个人不得对外提供、泄露，不得用于统计以外的目的”。

Confidentiality: According to Article 25 of Chapter 3 of the Statistics Law of the People's Republic of China, "Information obtained in statistical surveys that can identify or infer the identity of individual statistical survey subjects shall not be provided or disclosed to the public by any unit or individual, and shall not be used for purposes other than statistical purposes".

您好！这是一份关于“社会公众对器官捐献的态度、动机及影响因素”的统计调查问卷，希望能够得到您的参与。十分感谢您在百忙之中抽出时间参与我们的调查，谢谢！

Hello! This is a statistical questionnaire about "public attitude, motivation and influencing factors towards organ donation". We look forward to your participation. Thank you for your time and cooperation.

对问卷中问题的回答，没有对错之分，您只要根据平时的想法和实际情况回答就行。对于您的回答，我们将按照《中华人民共和国统计法》第一章第九条和第三章第二十五条的规定，对您所提供的所有信息绝对严格保密，并且只用于统计分析，请您不要有任何顾虑。我们在以后的科学研究、政策分析以及观点评论中发布的是大量问卷的信息汇总，而不是您个人、家庭的具体信息，不会造成您个人、家庭信息的泄漏。请您放心。

There is no right or wrong answer to the questions in the questionnaire; you just need to answer according to your usual thoughts and the actual situation. All information provided by you will be treated as strictly confidential and will only be used for statistical analysis by the provisions of Chapter 1, Article 9 and Chapter 3, Article 25 of the Statistics Law of the People's Republic of China. What we publish in our future scientific research, policy analysis, and opinion commentary is a summary of information from a large number of questionnaires, not specific information about you personally or your family, and will not result in any leakage of your personal or family information. Please do not worry.

1. 您的户口性质 [单选题]
   Household registration type [multiple choice]

○城市 Urban

○农村 Rural

2. 你的性别 [单选题]
   Gender [multiple choice]

○男 Male
○女 Female

3. 您的年龄 [单选题]
   Age [multiple choice]

○18 岁以下 under 18 years old
○18~30 岁 8-30 years old
○31~40 岁 31-40 years old
○41~50 岁 41-50 years old
○51~60 岁 51-60 years old
○60 岁以上 over 60 years old

4. 您的受教育程度 [单选题]
   Education level [multiple choice]

○初中及以下学历 junior high school and below
○高中及中专学历 high school and technical secondary school degree
○本科及大专学历 bachelor and college degree
○研究生及以上学历 postgraduate and above

5. 你是否知道器官捐献 [单选题]
   Do you know about organ donation [multiple choice]

○是 Yes
○否 No

6. 您是否有宗教信仰？ [单选题]
   Do you have a religious belief? [multiple choice]

○是 Yes
○否 No

7. 您知道我国颁布的《人体器官移植条例》这项法规吗 [单选题]
   Do you know the laws and regulations on human organ transplantation in China? [multiple choice]

○知道 Yes
○不知道 No

依赖于第 **5** 题第 1 个选项
If you choose the first option in question 5, please answer.

8. 您通过什么途径知道器官捐赠？ [多选题]
By what means do you know about organ donation? [one or more options can be chosen]
□书报杂志 books and magazines
□广播电视 radio and television
□互联网 internet
□宣传手册和海报 brochures and posters
□学校教育 school education
□亲朋好友 relatives and friends
□医护人员 medical staff
□其他 others

依赖于第 **5** 题第 1 个选项
If you choose the first option in question 5, please answer.

9. 由于供体器官缺乏，许多需要接受器官移植的患者在等待中死去，您是否知道这个情况？ [单选题]
Do you know that China is a massive lack of organ donation? [multiple choice]
○是 Yes
○否 No

10. 您是否清楚目前移植器官的来源？ [单选题] *
Do you know the current sources of transplanted organs? [multiple choice]
○是 Yes
○否 No

11. 您觉得哪种判断人死亡的指标更合理？ [单选题] *
Which index do you think is more reasonable to judge a person's death? [multiple choice]
○心脏死亡 cardiac death
○脑死亡 brain death
○脑心双死亡 cardiac death and brain death
○不清楚 unclear

12. 您是否愿意在身后捐献器官？ [单选题] *

Are you willing to donate organs after death? [multiple choice]

○愿意 Yes

○不愿意 No

13. 您是否填过捐献器官遗体的报名登记表？ [单选题] *

Have you filled in the organ donation registration form? [multiple choice]

○是 Yes

○否 No

依赖于第 **12** 题第 1 个选项

If you choose the first option in question 12, please answer.

14. 您愿意捐献哪些器官？ [多选题] *

What organs are you willing to donate? [one or more options can be chosen]

□眼角膜 Cornea

□心脏 heart

□肝脏 liver

□肾脏 kidney

□皮肤 skin

□骨髓 bone marrow

□遗体 remains (of the dead)

□其他 others ____________

依赖于第 **12** 题第 1 个选项

If you choose the first option in question 12, please answer.

15. 您希望捐献的器官将用于何处？ [多选题] *

What do you want the donated organs to be used for? [one or more options can be chosen]

□医学教学 medical teaching

□医学解剖研究 medical anatomy research

□临床移植 clinical transplantation

□其他 others ____________

依赖于第 **12** 题第 1 个选项

If you choose the first option in question 12, please answer.

16. 你觉得您的器官捐献意愿中受“从容面对死亡的心态”这一想法的影响程度是怎样的？（分数 1-5 分，影响程度由弱到强）[单选题]
To what extent have you been influenced by the idea of “calmly facing death”? (Score 1-5, from weak to strong) [multiple choice]

○1 ○2 ○3 ○4 ○5

依赖于第 **12** 题第 1 个选项
If you choose the first option in question 12, please answer.

17. 你觉得您的器官捐献意愿中受“救助他人，实现自身最后的价值”这一想法的影响程度是怎样的？（分数 1-5 分，影响程度由弱到强）[单选题]
To what extent have you been influenced by the idea of “helping others and realising your ultimate value”? (Score 1-5, from weak to strong) [multiple choice]

○1 ○2 ○3 ○4 ○5

依赖于第 **12** 题第 1 个选项
If you choose the first option in question 12, please answer.

18. 你觉得您的器官捐献意愿中受“赋予他人生命的同时延续自己的生命”这一想法的影响程度是怎样的？（分数 1-5 分，影响程度由弱到强）[单选题]
To what extent have you been influenced by the idea of “helping others and realising your ultimate value”? (Score 1-5, from weak to strong) [multiple choice]

○1 ○2 ○3 ○4 ○5

依赖于第 **12** 题第 1 个选项
If you choose the first option in question 12, please answer.

19. 你觉得您的器官捐献意愿中受“推动医疗事业发展”这一想法的影响程度是怎样的？（分数 1-5 分，影响程度由弱到强）[单选题] *
To what extent have you been influenced by the idea of “promoting the development of medical care”? (Score 1-5, from weak to strong) [multiple choice]

○1 ○2 ○3 ○4 ○5

依赖于第 **12** 题第 1 个选项
If you choose the first option in question 12, please answer.

20. 你觉得您的器官捐献意愿中受“家人朋友对器官捐献的支持”这一想法的影响程度是怎样的？（分数 1-5 分，影响程度由弱到强）[单选题]
To what extent have you been influenced by the idea of “support from family and friends for organ donation”? (Score 1-5, from weak to strong) [multiple choice]

○1 ○2 ○3 ○4 ○5

依赖于第 **12** 题第 1 个选项
If you choose the first option in question 12, please answer.

21. 你觉得您的器官捐献意愿中受“国家对器官捐献的宣传教育”这一想法的影响程度是怎样的？（分数 1-5 分，影响程度由弱到强）[单选题]
To what extent have you been influenced by the idea of “national publicity and education on organ donation”? (Score 1-5, from weak to strong) [multiple choice]

○1 ○2 ○3 ○4 ○5

依赖于第 **12** 题第 1 个选项
If you choose the first option in question 12, please answer.

22. 你觉得您的器官捐献意愿中受“社会对奉献精神倡导”这一想法的影响程度是怎样的？（分数 1-5 分，影响程度由弱到强）[单选题]
To what extent have you been influenced by the idea of “social advocacy and dedication”? (Score 1-5, from weak to strong) [multiple choice]

○1 ○2 ○3 ○4 ○5

依赖于第 **12** 题第 1 个选项
If you choose the first option in question 12, please answer.

23. 你觉得您的器官捐献意愿中受“对器官捐献流程的认知水平”这一想法的影响程度是怎样的？（分数 1-5 分，影响程度由弱到强）[单选题]
To what extent have you been influenced by the idea of “understanding level of organ donation process” (Score 1-5, from weak to strong) [multiple choice]

○1 ○2 ○3 ○4 ○5

依赖于第 **12** 题第 1 个选项
If you choose the first option in question 12, please answer.

24. 你觉得您的器官捐献意愿中受“对器官捐献法律法规的认知水平”这一想法的影响程度是怎样的？（分数 1-5 分，影响程度由弱到强） [单选题]
To what extent have you been influenced by the idea of “understanding the level of laws and regulations on organ donation”? (Score 1-5, from weak to strong) [multiple choice]

○1 ○2 ○3 ○4 ○5

依赖于第 12 题第 1 个选项
If you choose the first option in question 12, please answer.

25. 你觉得您的器官捐献意愿中受“对捐献器官如何使用的认知水平”这一想法的影响程度是怎样的？（分数 1-5 分，影响程度由弱到强） [单选题]
To what extent have you been influenced by the “understanding level about how donated organs are used”? (Score 1-5, from weak to strong) [multiple choice]

○1 ○2 ○3 ○4 ○5

依赖于第 12 题第 1 个选项
If you choose the first option in question 12, please answer.

26. 你觉得您的器官捐献意愿中“捐助者可以获得优先受捐权”这一想法的影响程度是怎样的？（分数 1-5 分，影响程度由弱到强） [单选题]
To what extent have you been influenced by the idea that “donors can get the priority right to receive organ donations”? (Score 1-5, from weak to strong) [multiple choice]

○1 ○2 ○3 ○4 ○5

依赖于第 12 题第 1 个选项
If you choose the first option in question 12, please answer.

27. 你觉得您的器官捐献意愿中“家属可以获得人文关怀和人道救助”这一想法的影响程度是怎样的？（分数 1-5 分，影响程度由弱到强） [单选题]
To what extent have you been influenced by the idea that “family members of donors can receive humanistic care and humanitarian assistance”? (Score 1-5, from weak to strong) [multiple choice]

○1 ○2 ○3 ○4 ○5

依赖于第 12 题第 2 个选项
If you choose the second option in question 12, please answer.

28. 你觉得您的器官捐献意愿中“对生死问题的回避态度”这一想法的影响程度是怎样的？（分数 1-5 分，影响程度由弱到强）[单选题]
To what extent have you been influenced by the idea of “the avoidance of life and death issues”? (Score 1-5, from weak to strong) [multiple choice]

○1 ○2 ○3 ○4 ○5

依赖于第 **12** 题第 2 个选项
If you choose the second option in question 12, please answer.

29. 你觉得您的器官捐献意愿中“保留全尸”的传统观念”这一想法的影响程度是怎样的？（分数 1-5 分，影响程度由弱到强）[单选题]
To what extent have you been influenced by the traditional concept of “keeping the whole corpse”? (Score 1-5, from weak to strong) [multiple choice]

○1 ○2 ○3 ○4 ○5

依赖于第 **12** 题第 2 个选项
If you choose the second option in question 12, please answer.

30. 你觉得您的器官捐献意愿中“家人不同意进行器官捐献”这一想法的影响程度是怎样的？（分数 1-5 分，影响程度由弱到强）[单选题]
To what extent have you been influenced by the idea that “family members don't agree to organ donation”? (Score 1-5, from weak to strong) [multiple choice]

○1 ○2 ○3 ○4 ○5

依赖于第 **12** 题第 2 个选项
If you choose the second option in question 12, please answer.

31. 你觉得您的器官捐献意愿中“家人不了解器官捐献法律法规”这一想法的影响程度是怎样的？（分数 1-5 分，影响程度由弱到强）[单选题]
To what extent have you been influenced by the idea that “family members don't know the laws and regulations of organ donation”? (Score 1-5, from weak to strong) [multiple choice]

○1 ○2 ○3 ○4 ○5

依赖于第 **12** 题第 2 个选项
If you choose the second option in question 12, please answer.

32. 你觉得您的器官捐献意愿中“媒体宣传力度不足”这一想法的影响程度是怎样的？（分数 1-5 分，影响程度由弱到强）[单选题]
To what extent have you been influenced by the idea of “insufficient media publicity”? (Score 1-5, from weak to strong) [multiple choice]

○1 ○2 ○3 ○4 ○5

依赖于第 **12** 题第 2 个选项
If you choose the second option in question 12, please answer.

33. 你觉得您的器官捐献意愿中“社会阶级不平等”这一想法的影响程度是怎样的？（分数 1-5 分，影响程度由弱到强）[单选题]
To what extent have you been influenced by the idea of “social class inequality”? (Score 1-5, from weak to strong) [multiple choice]

○1 ○2 ○3 ○4 ○5

依赖于第 **12** 题第 2 个选项
If you choose the second option in question 12, please answer.

34. 你觉得您的器官捐献意愿中“对死亡标准不清楚”这一想法的影响程度是怎样的？（分数 1-5 分，影响程度由弱到强）[单选题]
To what extent have you been influenced by the idea that “the standard of death is unclear”? (Score 1-5, from weak to strong) [multiple choice]

○1 ○2 ○3 ○4 ○5

依赖于第 **12** 题第 2 个选项
If you choose the second option in question 12, please answer.

35. 你觉得您的器官捐献意愿中“对器官捐献的法律条文不了解”这一想法的影响程度是怎样的？（分数 1-5 分，影响程度由弱到强）[单选题]
To what extent have you been influenced by the idea that “I do not know the legal provisions of organ donation”? (Score 1-5, from weak to strong) [multiple choice]

○1 ○2 ○3 ○4 ○5

依赖于第 **12** 题第 2 个选项
If you choose the second option in question 12, please answer.

36. 你觉得您的器官捐献意愿中“担忧捐献体系的可信度”这一想法的影响程度是怎样的？（分数 1-5 分，影响程度由弱到强）[单选题]
To what extent have you been influenced by the idea that you “worry about the credibility of the donation system”? (Score 1-5, from weak to strong) [multiple choice]

○1 ○2 ○3 ○4 ○5

依赖于第 12 题第 2 个选项
If you choose the second option in question 12, please answer.

37. 你觉得您的器官捐献意愿中“担忧器官被滥用”这一想法的影响程度是怎样的？（分数 1-5 分，影响程度由弱到强）[单选题]
To what extent have you been influenced by the idea that you “worry about organ abuse”? (Score 1-5, from weak to strong) [multiple choice]

○1 ○2 ○3 ○4 ○5

依赖于第 12 题第 2 个选项
If you choose the second option in question 12, please answer.

37. 您认为该如何化解公众对器官捐献的不信任感？[多选题]
How do you think public distrust of organ donation can be addressed? [one or more options can be chosen]

□建立公开透明的程序 establish an open and transparent process
□完善制度来监督捐献行为 improve the system to monitor donations
□通过完善立法来保护捐献者的权益 improve legislation to protect the rights and interests of donors
□其他 others__________________

38. 您认为怎样才能提高器官捐献率？[多选题]
What do you think can be done to improve organ donation rates? [one or more options can be chosen]

□加大宣传倡导力度，提升意识，加深了解 more publicity and advocacy efforts to raise awareness and deepen people's understanding of organ donation
□给予物质补偿和精神嘉奖 give material compensation and spiritual awards
□健全器官捐献法律，加快相关法制建设 improve the law of organ donation and speed up the construction of the relevant legal system.
□促进器官捐献流程规范化 promote standardisation of the organ donation process.
□医疗机构透明化，降低公众不信任感 enhance the transparency of the operation of medical institutions and increase public trust
□加强家庭、学校、政府对于器官捐献的教育 increase education on organ donation

□其他 others ________________

39. 您是否支持申领驾照时进行器官捐献登记？ [单选题]
Do you support organ donation registration when applying for a driver's license? [multiple choice]
○支持 Yes
○反对 No
○无所谓 neutral

40. 对于同意并签订相关器官遗体捐献协议的人，如果对其本人和亲属实行物质补偿（如优先获得器官移植、优先享有医疗保障等），您是否支持这一做法？ [单选题]
Do you support the practice that if people agree and sign the relevant organ donation agreement, material compensation shall be given to themselves and their relatives (such as giving priority to organ transplantation and medical insurance, etc.)? [multiple choice]
○支持 Yes
○反对 No
○无所谓 neutral

41. 器官移植作为新兴的医学技术，您认为其在中国的发展前景如何？ [单选题] *
As a new medical technology, what do you think is the development prospect of organ transplantation in China? [multiple choice]
○没有前景，人们受到传统观念的束缚太过严重了 There is no prospect, and people are bound by traditional ideas too seriously.
○举步维艰，器官来源匮乏 It is struggling with a lack of organ sources.
○虽然艰难，但是在不久的将来可能会兴起 Although it is difficult, it may rise shortly.
○随着科技水平的发展和人们思想水平的解放，该技术一定会繁荣发展 With the development of science and technology and the understanding of people, this technology will surely prosper and develop.
○其他 Others
